# Application of Holistic Nursing in Uremic Patients with Hematodialysis Related Malnutrition

**Published:** 2017-04

**Authors:** Huifang ZHANG, Chongting LIN, Songbo YUAN, Qinghua WANG, Jiangcheng YANG

**Affiliations:** 1. Hemodialysis Room, Yantaishan Hospital, Yantai, Shandong, China; 2. Administrative Office, Binzhou Medical University, Yantai, Shandong, China; 3. School of Nursing, Binzhou Medical University, Yantai, Shandong, China; 4. Dept. of Nephrology, Haiyang Hospital of Traditional Chinese Medicine, Haiyang, Shandong, China

**Keywords:** Holistic nursing, Uremia, Hematodialysis, Moderate and severe malnutrition

## Abstract

**Background:**

We aimed to observe the effect of holistic nursing on patients undergoing hematodialysis for uremia who simultaneously were suffering from moderate to severe malnutrition.

**Methods:**

Eighty patients with uremia on maintenance hematodialysis with malnutrition between June 2014 and June 2015 from Yantaishan Hospital, Yantai, Shandong, China were included and equally and randomly were divided into the control group (n=43) and observation group (n=43). Routine nursing was used in the control group while holistic nursing was used in observation group (before, during and after dialysis) and the clinical effects in the two groups were compared after 3 months.

**Results:**

At follow-up visits, serum creatinine and urea nitrogen levels of the patients in the two groups were decreased, whereas hemoglobin and albumin levels were increased. In addition, these improvements were greater in the observation group and the differences were statistically significant (*P*<0.05). Furthermore, during follow-up visits, MQSGA and MIS scores of the two groups were lower and the scores of the observation group were lower than those in the control group were, and the differences were statistically significant (*P*<0.05).

**Conclusion:**

Holistic nursing is able to improve significantly malnutrition in patients with uremia on hematodialysis.

## Introduction

Maintenance hematodialysis (MHD) is an important substitution therapy for chronic end-stage renal disease (ESRD). Although it can prolong survival time, there are complications associated with MHD, a common one among which is malnutrition. MHD markedly aggravated malnutrition caused by primary uremia (1). It was shown in a joint evaluation by six dialysis centers in Europe and America (2) that 33% of patients on MHD experience mild to moderate malnutrition, while 6–8% present with severe malnutrition. Furthermore, in China the incidence rates of the different degrees of malnutrition are higher than in other countries (3).

Uremia and malnutrition are closely related to inflammation and atherosclerosis, and influences quality of life and long-term survival rate (4), which might be caused by dialysis itself and incorrect dietary intake (5). Since MHD treatment is long term and mainly based outside of a hospital setting, reasonable and correct nursing interventions are very important (6). Patients with uremia usually exhibit very poor residual renal function, and a variety of complications, such as malnutrition, severe anemia, infection, dynsfunction of the heart, lung and liver and other organs, among other problems (7). The number of patients with uremia on MHD has gradually increased in recent years (30%–60% in 2016). In addition to enhancing MHD nursing, it is also critical to focus on nutrition, immune function, and complications in these patients (8).

Holistic nursing is a novel method of nursing, which systematizes clinical nursing and nursing management, with knowledge of modern nursing as the guiding factor based on modern nursing practices (9). The goal of holistic nursing is to provide optimal care based on the physical, psychological, social, cultural and spiritual requirements of the patients (10).

This present study analyzed the application of holistic nursing in patients on MHD associated moderate or severe malnutrition.

## Materials and Methods

### Patients

A total of 86 uremic patients with moderate or severe malnutrition treated by MHD in Yantaishan Hospital from June 2014 to June 2015 were enrolled in this study and equally and randomly divided into the control group (n=43) and observation group (n=43). The inclusion criteria included: 1. At least 3 dialysis treatments of 2 h per week ; 2. Hemoglobin ≤ 90 g/L and albumin ≤ 30g/L; 3. Patient compliance with complete medical history. The exclusion criteria included: 1. Incidence of severe complications during dialysis that required urgent treatment such as infection, heart failure and shock or severe malnutrition that required transfusion or albumin supplementation; 2. The patient was simultaneously part of another study; 3. Patient quit the study while in progress or was not followed-up .

This study obtained approved consent from patients and their family members. Ethics Committee of Yantaishan Hospital approved the study. Based on their order of hospitalization, patients were randomly divided into the control and observation group. For the control group, there were 25 males and 18 females. Their ages were between 52–74 years old and the average age was 66.3±12.4 years. Dialysis time was 1 week to 3 months and the median time was 1 month. Serum creatinine levels were 654–923 μmol/L and the average was 754.6±82.9 μmol/L. The urea nitrogen levels were 8.5–12.3 mmol/L, the average was 10.2±3.3 mmol/L. Hemoglobin levels were 56–82 g/L, the average was 70.5±13.4 g/L. Albumin levels were 20–28 g/L, and the average was 23.2±5.6 g/L. For the observation group, there were 24 males and 19 females and their ages were between 50–73 years old, with average age of 66.2±11.5 years. Dialysis time was 2 weeks to 3.5 months and the median time was 1.3 months. Serum creatinine levels were 667–1032 μmol/L and the average was 782.9±76.6 μmol/L. Urea nitrogen levels were 8.6–14.3 mmol/L and the average was 10.5±3.6 mmol/L. Hemoglobin levels were 53–83 g/L and average was 70.2±14.2 g/L. Albumin levels were 21–29 g/L and the average was 23.5±5.3 g/L. Differences between the two groups in terms of these baseline parameters were not statistically significant (*P*>0.05).

### Nursing methods

Patients in the two groups were treated by standard MHD protocols and nursing procedures and were taken care of by the same clinical and nursing team. Routine nursing was used in the control group, which included matters requiring attention at home during dialysis, and ensuring patients were provided a reasonable diet, proper exercise and enough sleep. During dialysis, vital signs were observed closely and dialysis conditions were recorded.

Holistic nursing was applied to patients in the observation group at 3 stages: before, during and after dialysis. Patients were monitored for any adverse reactions during dialysis, recent diet, reexaminations of blood for liver and kidney function when necessary, and dialysis parameters for proper adjustment. The dialysis time and drug dosages were adjusted based on the nutritional status of patients. Dialysis itself is able to promote catabolism, proteolysis and reduce protein synthesis. For each dialysis treatment, 5–8 g amino acid, 5 g peptide and 20–30 g sugar will be lost. The application of heparin is also known to promote lipolysis. Meanwhile, insufficient dialysis will also cause adverse effects such as inappetence, toxin retention, acidosis, reduced intake of protein and other nutrient substances and increased catabolism. Arteriovenous fistula (AVF) was checked carefully for presence of obstructions. Inadequate nursing of internal fistula are known to influence the effects of dialysis, increase pain, cause fear related to dialysis and aggravate malnutrition.

Blood vessel protection before needle insertion of internal fistula was done appropriately in order to prevent obstruction caused by poor blood flow. The condition of fistula was closely checked and all related issues were handled in timely manner. Operators performed with strict aseptic technique and had good command of the puncture technique, thus preventing errhysis, hematoma formation, pseudoaneurysm and infection.

Following dialysis, proper diet, lifestyle and psychological management are critical for the quality of life of patients. Thus, strengthening patient knowledge on proper nutrition was achieved by providing lectures and organizing groups designed to spread health knowledge on a bi-weekly basis. The topics covered included causes of MHD-related malnutrition, clinical manifestation, and complications in order to help patients be made aware of the harm that can arise due to malnutrition and the significance of diet management, especially for new dialysis patients, helping them to adapt. Videos, brochures and oral explanations were offered to introduce knowledge of nutrition and dialysis in detail, including information on basic principles, purpose, food composition, restriction of water, sodium, potassium and phosphorus to help patients choose and adjust their diets. The patients’ family members were educated as well about the ingredients of common food items and calculations regarding food intake. Appropriate nursing measures were established based on the specific conditions of each patient, such as choosing food properly, changing cooking method, eating less but more frequently, encouraging patients to exercise properly and promoting communication between patients and their families to create a positive atmosphere. Specialized nurses were there to record and make calculations based on diet, evaluate patient compliance with diet and determine reasons of insufficient intake in order to improve malnutrition effectively.

We applied additional forms of education such as sharing personal experience, individual instruction, group explanations and discussions and establishing strong relationships between patients and nurses to improve nursing attitude of family members and strengthen the support system, thereby increasing patient compliance and enhancing effectiveness of transmitting nutritional knowledge.

### Observation indexes

Median time of follow-up visit was 12 months. Changes of serum creatinine, urea nitrogen, hemoglobin and albumin were compared. Modified Quantitative Subjective Global Assessment (MQSGA) and Malnutrition Inflammation Score (MIS) were used for questionnaire survey. MQSGA is a quantitative scoring system, which mainly scores weight changes, dietary intake changes, gastrointestinal symptoms, functional status changes, complications, and subcutaneous fat and muscle wasting. The score range for each part is 1 point (normal) to 5 points (severe), and the total score is between 7 (normal nutrition) to 35 points (severe malnutrition). The possibility of malnutrition is higher if the score is higher. Conditions of related medical history were obtained through questionnaire survey and physical examination. The MIS scoring system consisted of 10 parts, 0–3 points for each part and the higher the score, the worse the nutritional status.

### Statistical analysis

SPSS 19.0 (Chicago, IL, USA) was used for data input and analysis. Quantitative data is expressed as mean value ± standard deviation and comparisons between groups were by *t*-test. Qualitative data is expressed by case or percentage (%) and comparisons between groups were by χ^2^ test. *P*<0.05 was taken as statistically significant.

## Results

### Changes of serum creatinine, urea nitrogen, hemoglobin and albumin

Upon follow-up visits, serum creatinine and urea nitrogen levels of patients in the two groups were lower while hemoglobin and albumin levels increased. The improved conditions of patients in the observation group were greater than in the control group and the differences were statistically significant (*P*<0.05) (Table 1).

**Table 1:** Follow-up on changes of serum creatinine, urea nitrogen, hemoglobin and albumin

**Group**	**Creatinine (μmol/L)**	**Urea nitrogen (mmol/L)**	**Hemoglobin (g/L)**	**Albumin (g/L)**
Control group	526.3±76.9	8.5±2.4±2.4	82.4±6.5	28.2±3.3
Observation group	421.7±54.2	6.3±2.7±2.3	93.3±6.6	35.1±3.5
*t*	6.957	5.438	5.827	6.102
*P*	0.007	0.013	0.010	0.009

### MQSGA and MIS scoring

There were no statistically significant differences in MQSGA or MIS score between the two groups (*P*>0.05). However, at follow-up visits, MQSGA and MIS scores lowered significantly. In addition, they were lower in the observation group compared to the control group, and the differences were of statistical significance (*P*<0.05) (Table 2).

**Table 2:** MQSGA and MIS scoring

**Group**	**MQSGA at grouping**	**MQSGA in follow-up**	**MIS at grouping**	**MIS in follow-up**
Control group	26.6±5.5	20.2±4.8	24.7±3.6	18.9±3.2
Observation group	26.7±5.2	15.4±4.4	25.2±3.3	13.2±2.8
*t*	0.624	5.418	0.392	5.936
*P*	0.825	0.015	0.401	0.007

## Discussion

There are several common causes of malnutrition in hematodialysis patients:

- Insufficient nutrient intake caused by anorexia and lack of nutritional knowledge (11). Factors such as urotoxin retention, acid, gastrointestinal dysfunction, medication, anorexia from mental and social factors, nausea and vomiting can result in dietary restriction which will lower protein and energy intake below body requirements. Hematodialysis itself can also cause nausea and vomiting (11). Dialysis delays gastric emptying and therefore can aggravate anorexia. There is evidence that a proportion of patients lack knowledge about nutritional intake during dialysis, while others are used to their previous non-dialysis methods and are afraid to increase protein intake (11). Energy intake is insufficient due to dietary restrictions of sugar and fat and increasing levels of blood lipids will cause usage rate of protein to decrease. Nutritional intake is not ideal due to lack of knowledge.
- Malnutrition caused by uremia and dialysis (12). Renal diseases cause lowered immune function along with chronic diseases such as chronic gastroenteritis, and heart failure (12). Acute diseases such as upper respiratory infection, which result in deterioration of negative nitrogen balance and nutritional conditions, increase catabolism and reduce protein and fat storage. According to a previous study (13), the appetites of hematodialysis patients during treatment was significantly lower than in non-dialysis and healthy controls, about 50% of MHD patients had insufficient protein intake and 90% had insufficient energy intake. The biocompatibility and nutritional status of dialysis membranes are closely related. If the biocompatibility of dialysis membranes are poor, then catabolism of protein will be accelerated. The biocompatibility of polysulfone membranes is better than cuprophane membranes, therefore polysulfone is able to improve the malnutrition of patients undergoing hemodialysis to some degree (13). In addition, factors such as oxidative stress of the dialysis membrane and contamination of the dialysis solution will cause microinflammation, resulting in a vicious circle of malnutrition.
- Influences of metabolic acidosis and endocrine dysfunction (14). Metabolic acidosis will promote proteolysis and oxidation of branched chain amino acids. Many factors will promote proteolysis and reduce protein synthesis such as hyposecretion and insufficient supplement of hemopoietin, insulin resistance, insensitivity to growth hormones and insulin-like growth factor, hyperinsulinemia and hyperparathyroidism.
- Influences of age and metal factors (15). Malnutrition is generally worse in older patients undergoing hematodialysis. MHD is a lifelong treatment, there are many complications at the late period, and the cost of treatment is high, which causes some patients to feel pessimistic. Among which, common mental disorders such as anxiety, irritability and depression will influence the development and prognosis of the disease and aggravate malnutrition.

Holistic nursing promotes a personalized and scientific approach to nursing based on causes of malnutrition in MHD and the conditions of patients, among which dietary management is a key factor. Diets should be managed as follows:

- Required caloric intake must be based on the nutritional conditions of patients. The minimum intake is >146.5 KJ/ (kg·d). Grains, meat, egg yolk, cream, milk and nut fruits are calorically dense foods (16).
- Necessary protein intake should be guaranteed. For patients receiving dialysis twice a week, it should be 1.0–1.2 g/ (kg·d). For dialysis three times a week, it should be 1.2–1.5 g/ (kg·d). With animal protein as the main source of amino acids and with no restriction on intake of vegetable protein, the body is able to effectively use ingested protein, maintain sufficient nutrient storage, and maintain an optimal nutritional state (17). However, since there is high potassium content in animal protein, hyperkalemia is a concern, which must also be prevented.
- Water intake should be based on daily urinary volume, dialysis frequency, dialysis time, edema and blood pressure (18). Patients should be informed about the importance of water and sodium intake restriction so that they will positively cooperate based on their treatment regimen and control it themselves to avoid heart failure and control weight increases within a 2 kg range during dialysis. Salt intake should be 3–5 g, and proper sodium intake restriction will avoid thirst and reduce water intake.
- Adjustments of electrolyte intake must be made (19). Daily potassium intake should be restricted to less than 2 g. Food containing high potassium includes beans, potato and winter bamboo shoots. Most hematodialysis patients present with low calcium and high phosphorus, therefore their phosphorus intake should be restricted and calcium intake should be increased. Daily calcium intake should be roughly 1–1.5 g. Foods such as beans, fungi and nut fruits contain high phosphorus, while dairy products, sesame and beans contain high calcium.

Limitations of this study should also be considered. Given that this research was conducted by the same nursing team, nursing procedure in observation group and control group cannot be completely separated. In addition, the sample of the present study was relatively small, thus, further studies were required to better support our results.

## Conclusion

In the group treated by holistic nursing, serum creatinine and urea nitrogen levels were significantly lowered while hemoglobin and albumin levels increased significantly. MQSGA and MIS scores were significantly lowered compared to the control group. Finally, holistic nursing is able to improve significantly malnutrition of uremic patients on hematodialysis.

## Ethical considerations

Ethical issues (Including plagiarism, informed consent, misconduct, data fabrication and/or falsification, double publication and/or submission, redundancy, etc.) have been completely observed by the authors.

## References

[1] Hasheminejad N Namdari M Mahmoodi MR Bahrampour A Azmandian J (2016). Association of Handgrip Strength with Malnutrition-Inflammation Score as an Assessment of Nutritional Status in Hemodialysis Patients. Iran J Kidney Dis, 10 (1):30–5. 26837679

[2] Kopple JD (2001). National Kidney Foundation K/DOQI clinical practice guidelines for nutrition in chronic renal failure. Am J Kidney Dis, 37(1 Suppl 2):S66–70. 11158865 10.1053/ajkd.2001.20748

[3] Xie LM Ge YY Huang X Zhang YQ Li JX (2015). Effects of fermentable dietary fiber supplementation on oxidative and inflammatory status inhemodialysis patients. Int J Clin Exp Med, 8(1):1363–9. 25785138 PMC4358593

[4] Kaysen GA (2000). Malnutrition and the acute-phase reaction in dialysis patients-how to measure and how to distinguish. Nephrol Dial Transplant, 15(10):1521–1524. 11007817 10.1093/ndt/15.10.1521

[5] Karavetian M Elzein H Rizk R Jibai R de Vries N (2016). Nutritional education for management of osteodystrophy: Impact on serum phosphorus, quality of life, and malnutrition. Hemodial Int, 20(3):432–40. 26843138 10.1111/hdi.12405

[6] Kargar JM Javadpour S Taheri L Poorgholami F (2015). Effect of Nurse-Led Telephone Follow ups (Tele-Nursing) on Depression, Anxiety and Stress in Hemodialysis Patients. Glob J Health Sci, 8(3):168–73. 26493429 10.5539/gjhs.v8n3p168 PMC4804080

[7] Formica M Marazzi F Tamagnone M (2014). Chronic uremia and palliative care. G Ital Nefrol, 31(2): gin/31.2.4.. 24777917

[8] Kovesdy CP Kalantar-Zadeh K (2016). Back to the future: restricted protein intake for conservative management of CKD, triple goals of renoprotection, uremia mitigation, and nutritional health. Int Urol Nephrol, 48(5):725–9. 26886110 10.1007/s11255-016-1224-0 PMC5061032

[9] Reid A (2015). Holistic Nursing, Reiki, & the Centle Art of Beekeeping. Beginnings, 35(6):22–4. 26867264

[10] Koithan M (2015). The Promise of Integrative Nursing. Creat Nurs, 21(4):193–9. 26731916 10.1891/1078-4535.21.4.193

[11] Santos PR Silveira Monteiro DL de Paula PH (2015). Dyspepsia is Associated with Low Protein and Caloric Intake among End-Stage Renal Disease Patients. Int J Vitam Nutr Res, 85(3–4):112–8. 26780390 10.1024/0300-9831/a000230

[12] Ren H Gong D Jia F Xu B Liu Z (2016). Sarcopenia in patients undergoing maintenance hemodialysis: incidence rate, risk factors and its effect on survival risk. Ren Fail, 38(3):364–71. 26738817 10.3109/0886022X.2015.1132173

[13] Rocco MV Paranandi L Burrowes JD (2002). Nutritional status in the HEMD study cohort at baseline. Hemodialysis. Am J Kidney Dis, 39(2): 245–256. 11840364 10.1053/ajkd.2002.30543

[14] Li W Zhang S (2015). Risk Factors of Parathyroid Dysfunction in Elderly Patients with Chronic Kidney Disease Undergoing Hemodialysis. Adv Clin Exp Med, 24(6):1007–12. 26771973 10.17219/acem/23439

[15] Finkelstein FO Wuerth D Finkelstein SH (2010). An approach to addressing depression in patients with chronic kidney disease. Blood Purif, 29: 121–4. 20093816 10.1159/000245637

[16] Shah A Bross R Shapiro BB Morrison G Kopple JD (2016). Dietary energy requirements in relatively healthy maintenance hemodialysis patients estimated from long-term metabolic studies. Am J Clin Nutr, 103(3):757–65. 26864370 10.3945/ajcn.115.112995 PMC4763489

[17] Beto JA Schury KA Bansal VK (2016). Strategies to promote adherence to nutritional advice in patients with chronic kidney disease: a narrative review and commentary. Int J Nephrol Renovasc Dis, 2;9:21–33. 26893578 10.2147/IJNRD.S76831 PMC4749088

[18] Yilmaz S Yildirim Y Taylan M (2016). The relationship of fluid overload as assessed by bioelectrical impedance analysis with pulmonary arterial hypertension in hemodialysis patients. Med Sci Monit, 22:488–494. 26874785 10.12659/MSM.896305 PMC4755666

[19] Haras MS ( 2015 ). Does hemodialysis dialysate potassium composition matter? Nephrol Nurs J, 42 : 577 –580. 26875233

